# A CRISPR/dCasX‐mediated transcriptional programming system for inhibiting the progression of bladder cancer cells by repressing c‐MYC or activating TP53

**DOI:** 10.1002/ctm2.537

**Published:** 2021-09-15

**Authors:** Congcong Cao, Lin Yao, Aolin Li, Quan Zhang, Zhenan Zhang, Xiaofei Wang, Ying Gan, Yuchen Liu, Qian Zhang

**Affiliations:** ^1^ Department of Urology Peking University First Hospital Beijing China; ^2^ Institute of Urology Peking University Beijing China; ^3^ National Urological Cancer Center Beijing China; ^4^ Beijing Key Laboratory of Urogenital Diseases (male) Molecular Diagnosis and Treatment Center Beijing China; ^5^ Guangdong Key Laboratory of Systems Biology and Synthetic Biology for Urogenital Tumors Shenzhen Institute of Translational Medicine, Shenzhen Second People's Hospital The First Affiliated Hospital of Shenzhen University Shenzhen China; ^6^ Guangdong and Shenzhen Key Laboratory of Male Reproductive Medicine and Genetics Institute of Urology Peking University Shenzhen Hospital, Shenzhen‐Peking University‐The Hong Kong University of Science and Technology Medical Center Shenzhen China

To the Editor:

The CRISPR‐associated catalytically inactive SpdCas9 and AsdCas12a proteins fused to effector domains, with distinct regulatory functions enables stable and efficient transcriptional repression or activation.^1^, ^2^, ^3^, ^4^ Recently, DpbCas12e protein (formerly CasX) from *Deltaproteobacteria* forms a distinct subtype of Class II type V CRISPR‐Cas effectors and can introduce programmable double‐stranded breaks in mammalian genomes.^5^ Here, we identify that fusions of the nuclease‐inactive dead CasX (dCasX) to the KRAB repressor (dCasX‐KRAB) or VPR activator (dCasX‐VPR) can silence or activate target gene expression in human cells.

To construct a robust and tunable gene repression system, we first created a gene encoding a human codon optimized dCasX fused with the repressive chromatin modifier domain named KRAB (Figure 1A, Figure S1, Tables S1 and S2). We also compared the transcriptional repression efficiency of dCasX‐KRAB to that of dCas9‐KRAB and dCas12a‐KRAB.^3^, ^6^ A green fluorescent protein gene (GFP) expression cassette was driven by a CMV promoter. The dCas9‐gRNA, dCas12a‐crRNA, and dCasX‐gRNA were designed to bind to their complementary targeted regions, which were located close to CMV promoter sequence (Figure 1A). The results showed that dCasX‐KRAB‐gRNA‐GFP expression was sufficient to silence GFP fluorescence and transcripts, but might be less effective than dCas9‐KRAB and dCas12a‐KRAB in transcriptional repression (Figure 1B–D, Figures S2 and S3). As there can be important differences in how endogenous and reporter genes are transcribed, we tested whether dCasX‐KRAB can silence endogenous human gene. Two target sequences were designed for the promoter region of oncogene c‐MYC (Figure 1E). Two days after co‐expression of dCasX‐KRAB with sgRNA‐NT (sgRNA nontargeted) or sgRNA‐c‐MYC in bladder cancer cells, repression of c‐MYC was observed, as measured by RT‐qPCR and Western blot, and stronger repression was achieved when a combination of two sgRNAs was expressed (Figure 1F, Figure S4). This observation suggested that our system could knockdown targeted gene expression by co‐expression of gRNA and dCasX‐KRAB in human cells.

**FIGURE 1:**
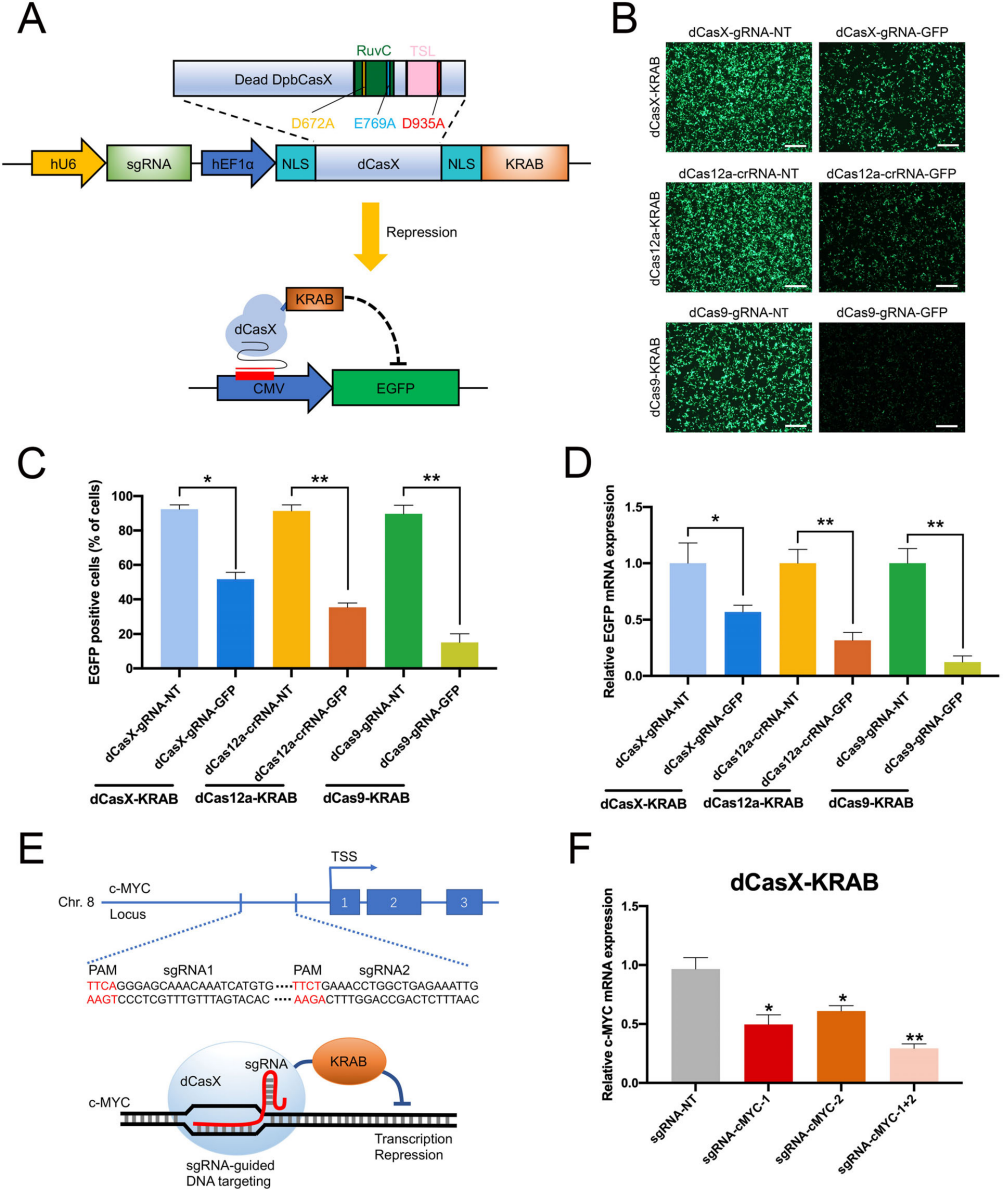
dCasX‐KRAB system leads to robust transcription repression in human cell lines. (A) Schematic diagram of the sgRNA‐guided dCasX‐KRAB gene repression system that consists of two parts: one part contains dCasX‐KRAB driven by a hEF1α promoter; another part contains sgRNA targeting the promoter of the gene of interest driven by the human U6 promoter. Upon cotransfection of the sgRNA‐guided dCasX‐KRAB and CMV‐EGFP plasmids, dCasX‐KRAB can repress the GFP transcription downstream of the CMV promoter. (B) dCas9, dCas12a, and dCasX‐KRAB‐based transcriptional repressors displayed RNA‐guided EGFP transcriptional repression, as detected by fluorescent microscopy in HEK293T cells. Representative images of the transfected cells are shown. gRNA‐NT and crRNA‐NT were used as control. Scale bar, 200 μm. (C) Flow cytometry analysis of GFP‐positive cells transfected with plasmids in (B). (D) RT‐qPCR analysis of GFP mRNA expression 2 days after being transfected with plasmids in (B). (E) c‐MYC sgRNA targeting sites were located at upstream of the c‐MYC transcription starting site (TSS); protospacer‐adjacent motif (PAM) sequences in red; blue boxes indicate exons. (F) c‐MYC mRNA expression level detected by RT‐qPCR after transfection using dCasX‐KRAB‐sgRNA‐c‐MYC or dCasX‐KRAB‐sgRNA‐NT control group in bladder cancer cells. *p < .05, **p < .01 by Student's *t*‐test

Furthermore, we examined the effect of repressed endogenous c‐MYC, a well‐studied oncogene that positively regulates cell proliferation,^7^ by using dCasX‐KRAB in bladder cancer cells via a set of functional assays. The cell proliferation assay indicated that dCasX‐KRAB targeting c‐MYC significantly reduced both 5637 and T24 bladder cancer cells growth (Figure 2A,B). Then the cell apoptosis was examined using caspase‐3/ELISA and it showed that the dCasX‐KRAB targeting c‐MYC significantly increased the cell apoptosis in both bladder cancer cells (Figure 2C). Finally, the wound‐healing assay indicated that dCasX‐KRAB targeting c‐MYC tended to attenuate migration of both bladder cancer cells (Figure 2D–F). To further explore potential applications of dCasX‐KRAB, we conducted in vivo experiments with subcutaneous tumor models. When c‐MYC was silenced by dCasX‐KRAB, volumes and weights of xenografted tumors were decreased compared with that of control group (Figure 2G–J). In addition, we observed adeno‐associated virus (AAV)‐vector‐mediated in vivo delivery of dCasX‐KRAB‐cMYC system could also ameliorate the growth of tumors (Figure S5). These results confirmed that targeted downregulation of oncogene by dCasX‐KRAB can inhibit the malignant phenotypes of bladder cancer.

**FIGURE 2:**
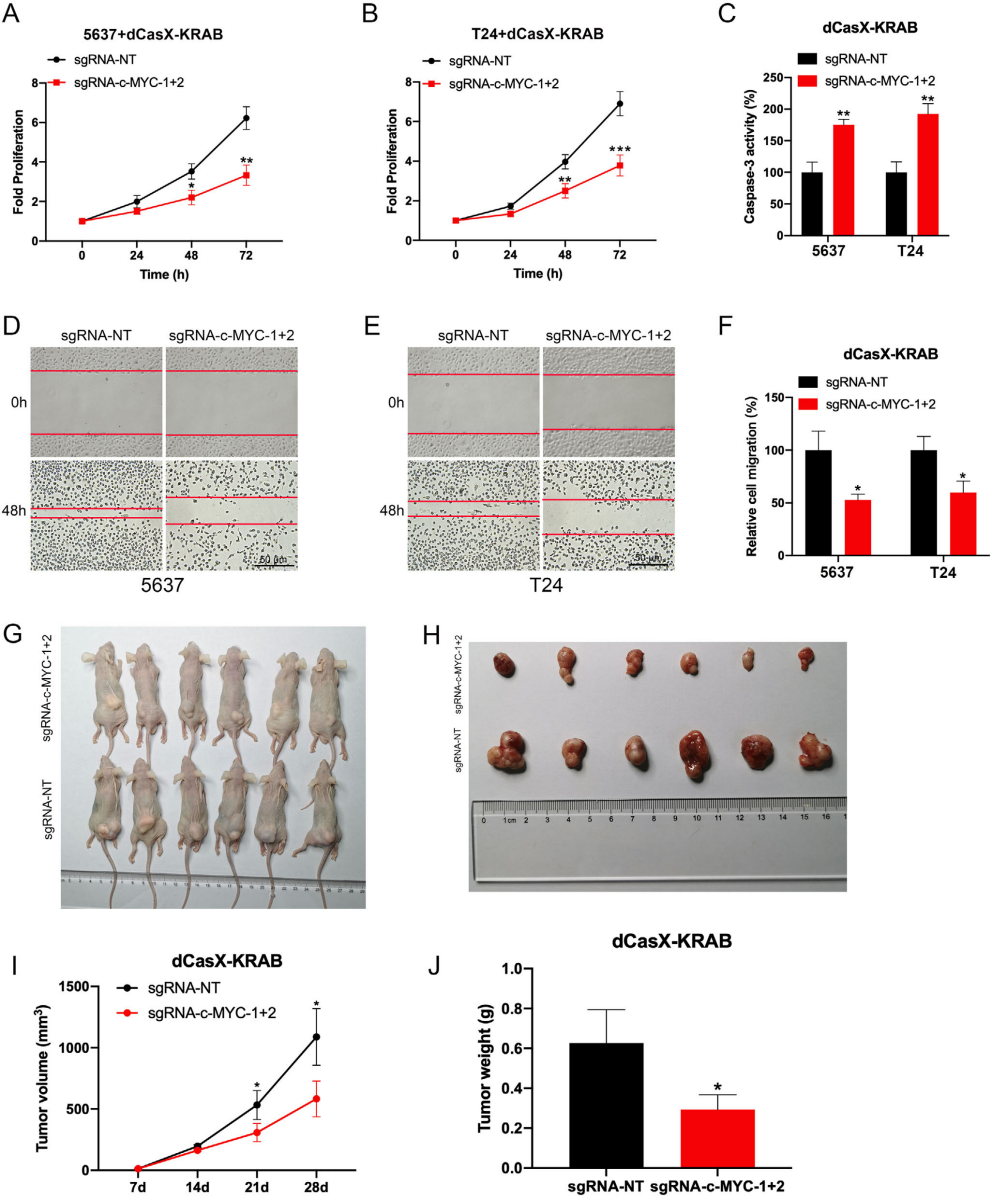
Inhibitory effect of dCasX‐KRAB‐repressed c‐MYC expression in bladder cancer cells. (A and B) CCK‐8 assay indicating the effect of dCasX‐KRAB‐repressed c‐MYC expression on 5637 and T24 bladder cancer cells proliferation. (C) Caspase‐3/ELISA suggesting the effect of dCasX‐KRAB‐repressed c‐MYC expression on 5637 and T24 cell apoptosis. (D–F) Wound‐healing assays were conducted to compare the migration capabilities of two bladder cancer cells after silencing of c‐MYC by dCasX‐KRAB. The difference in cell margin between 0 and 48 h showed the moving track of cells. The percentage of healed area was quantified (F). (G–J) Subcutaneous tumor model of bladder cancer cells with c‐MYC repression and corresponding negative control. Tumor volume and weight were measured at the indicated weeks after mice were transplanted. *p < .05, **p < .01, ***p < .001 by Student's *t*‐test

We reasoned that the CRISPRi platform provides a modular protein effector recruitment system, which may also be useful for gene activation when coupled with transcription activators. To test if we can activate gene expression in human cells with dCasX, we fused a hybrid VP64‐p65‐Rta (VPR) tripartite activator to dCasX (Figure 3A, Figure S1, Tables S1 and S2). We also compared the transcriptional activation efficiency of dCasX‐VPR to that of dCas9‐VPR and dCas12a‐VPR in HEK293T cells. The dCas9‐gRNA, dCas12a‐crRNA, and dCasX‐gRNA were designed to bind to sequences near the TATA‐box of the GFP reporter plasmid (Figure 3A). Two days following transfection, we observed that the dCasX‐VPR‐gRNA‐EGFP induced increases in GFP expression and the transcriptional activation efficiency of dCasX‐VPR might be stronger than that of dCas9‐VPR and dCas12a‐VPR (Figure 3B–D, Figures S6 and S7). This may be due to the enzymology of these three Cas nucleases or their unique biochemical/biophysical properties. Then we tested whether dCasX‐VPR could activate endogenous gene in human cells. For an endogenous gene target, we selected TP53, which is widely known as a tumor suppressor. Two sgRNAs were designed to bind to sequences near the transcription start site of the TP53 promoter (Figure 3E). Two days after transient transfection, the expression of each sgRNA resulted in strong activation of TP53 expression compared to the nontarget sgRNA control (Figure 3F, Figure S8). This result indicated that dCasX‐VPR system could be a useful tool for targeted gene activation in human cells.

**FIGURE 3:**
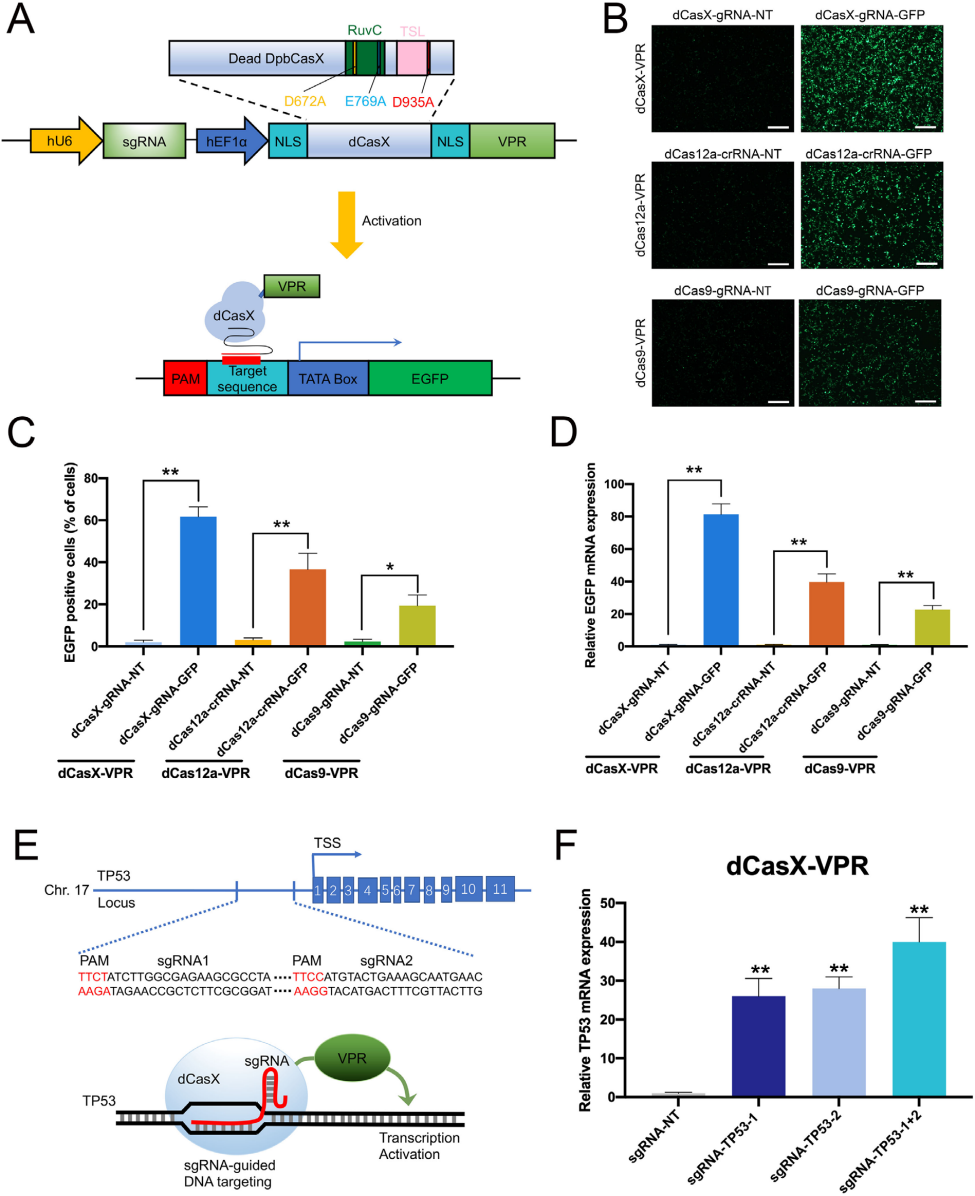
dCasX‐VPR can efficiently activate transcription in human cells. (A) Schematic diagram of the sgRNA‐guided dCasX‐VPR gene activation system that consists of two parts: one part contains dCasX‐VPR driven by a hEF1α promoter; another part contains sgRNA targeting the promoter of the gene of interest driven by the human U6 promoter. Upon cotransfection of the sgRNA‐guided dCasX‐VPR and TATA‐box‐GFP reporter, dCasX‐VPR can activate the GFP transcription downstream of the TATA‐box promoter. (B) dCas9, dCas12a, and dCasX‐VPR‐based transcriptional activators displayed RNA‐guided EGFP transcriptional activation, as detected by fluorescent microscopy in HEK293T cells. Representative images of the transfected cells are shown. gRNA‐NT and crRNA‐NT were used as control. Scale bar, 200 μm. (C) Flow cytometry analysis of GFP‐positive cells transfected with plasmids in (B). (D) RT‐qPCR analysis of GFP mRNA expression 2 days after transfected with plasmids in (B). (E) TP53 sgRNA targeting sites were located at upstream of the TP53 transcription starting site (TSS); protospacer‐adjacent motif (PAM) sequences in red; blue boxes indicate exons. (F) TP53 mRNA expression level detected after transfected with dCasX‐VPR‐sgRNA‐TP53 or dCasX‐VPR‐sgRNA‐NT control group in bladder cancer cells by RT‐qPCR. *p < .05, **p < .01 by Student's *t*‐test

The effect of TP53 expression activated by dCasX‐VPR was assessed on the bladder cancer cells. CCK‐8 assay results indicated that TP53 activation decreased 5637 and T24 bladder cancer cells proliferation (Figure 4A,B). Then, the dCasX‐VPR‐activated TP53 expression significantly promoted the cell apoptosis in both cell lines detected by caspase‐3/ELISA (Figure 4C). Finally, the wound‐healing assay indicated that dCasX‐VPR targeting TP53 tended to attenuate migration of both bladder cancer cells (Figure 4D–F). The effects of TP53 expression activated by dCasX‐VPR in vivo were further studied. We used TP53 activation or the control cells to generate a subcutaneous xenograft tumor mouse model. As expected, TP53 activation cells (sgRNA‐TP53‐1+2) showed smaller tumor volume and weight compared to the control cells (sgRNA‐NT) (Figure 4G–J). This was similar to the results of in vitro experiments in which a more significant tumor inhibition effect was observed with the increase of time. These results confirmed that targeted activation of TP53 by dCasX‐VPR can inhibit the progression of bladder cancer cells.

**FIGURE 4:**
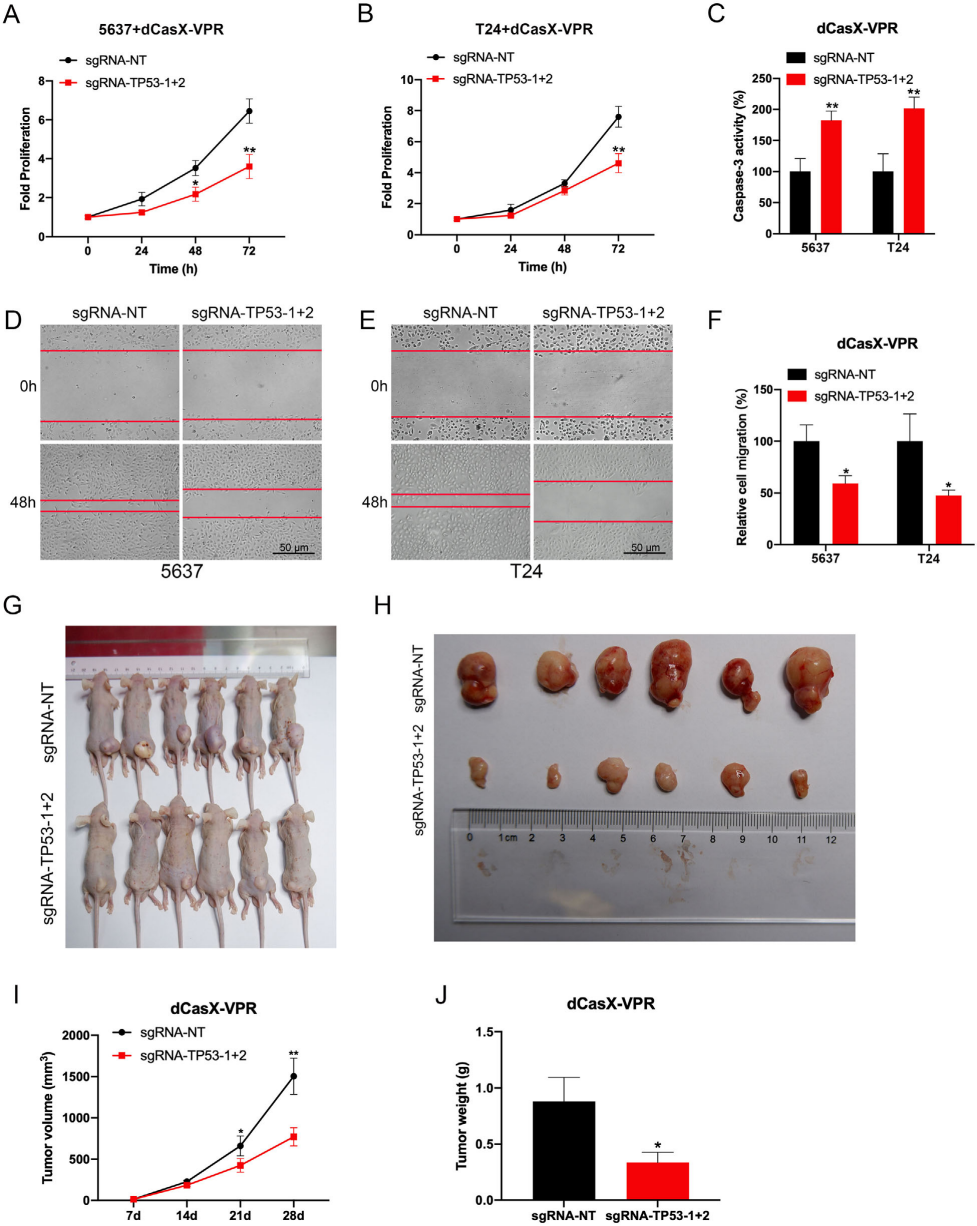
Targeting TP53 activation by dCasX‐VPR regulates bladder cancer cell activities. (A and B) CCK‐8 assay indicating the effect of dCasX‐VPR‐activated TP53 expression on 5637 and T24 bladder cancer cells proliferation. (C) Caspase‐3/ELISA suggesting the effect of dCasX‐VPR‐activated TP53 expression on 5637 and T24 cell apoptosis. (D–F) Wound‐healing assays were conducted to compare the migration capabilities of two bladder cancer cells after activation of TP53 by dCasX‐VPR. The difference in cell margin between 0 and 48 h showed the moving track of cells. The percentage of healed area was quantified (F). (G–J) Subcutaneous tumor model of bladder cancer cells with TP53 activation and corresponding negative control. Tumor volume and weight were measured at the indicated weeks after mice were transplanted. *p < .05, **p < .01 by Student's *t*‐test

Previous studies have shown that CasX is much smaller than Cas9, but is more efficient in genome editing.^5^ Based on our results, dCasX may be a more suitable candidate Cas protein for transcriptional activation and therapeutic delivery, although further tests should be done on more endogenous targets using the stably transfected cells (Figures S9 and S10).

In conclusion, the CRISPR/dCasX system can be used to screen for both loss‐of‐function and gain‐of‐function phenotypes in a modular format and may produce an anticaner effect when driven by cancer‐specific promoters.

## CONFLICT OF INTEREST

The authors declare that there is no conflict of interest that could be perceived as prejudicing the impartiality of the research reported.

## AUTHOR CONTRIBUTIONS

Qian Zhang, Yuchen Liu and Ying Gan were responsible for the study design and supervision and wrote the paper. Congcong Cao, Lin Yao and Aolin Li performed the experiments. Quan Zhang, Zhenan Zhang and Xiaofei Wang performed data analysis. All authors approved the final version of the manuscript.

## FUNDING INFORMATION

National Natural Science Foundation of China, Grant Numbers: 82072826, 81872088, 81972867, 81773257; Peking University Medicine Fund of Fostering Young Scholars' Scientific & Technological Innovation, Grant Number: BMU2020PYB028; Natural Science Foundation of Guangdong, Grant Number: 2018B030306023; Shenzhen Municipal Government of China, Grant Numbers: RCYX20200714114701035, JCYJ20180507184642475.

## Supporting information

SUPPORTING INFORMATION
