# Supplementary material for: A CRISPR/dCasX‐mediated transcriptional programming system for inhibiting the progression of bladder cancer cells by repressing c‐MYC or activating TP53
Source: Clin Transl Med. 2021 Sep 15;11(9):e537. doi: 10.1002/ctm2.537 (PMC8441141; doi:10.1002/ctm2.537)
Supplement: Supplementary file 1 — SUPPORTING INFORMATION [file CTM2-11-e537-s001.docx]

**Materials and Methods**

**﻿Plasmids Construction**

DNA encoding the human sequence-optimized dCasX nuclease harboring the inactivating D672A, E769A and D935A substitution was chemically synthesized and cloned into a plasmid containing a HEf1α promoter to yield plasmid dCasX–sgRNA. The dCasX sequence fused with the KRAB domain sequence was then inserted into the same backbone to form plasmid dCasX–KRAB–sgRNA. The dCasX–VPR–sgRNA construct was assembled by fusing dCasX with VPR at the N terminus. The plasmid maps are shown in Supplementary Fig. 1a–f. The original sgRNAs were designed using the online design tool “Cas-Designer” (http://www.rgenome.net/cas-designer/). The designed complimentary DNA (cDNA) sequence for each sgRNA was synthesized and inserted into the corresponding plasmid expressing both dCasX and crRNA. These plasmids were constructed by Syngentech Co., Ltd. (Beijing, China). The dCas9 and dCas12a relative plasmids were purchased from Addgene. The above plasmids and relative sequences were listed in **Supplementary Material.**

﻿**Cell Lines and Cell Culture**

﻿HEK 2923T (Human embryonic kidney cell line) and Hela cell were purchased from the Institute of Cell Research, Chinese Academy of Sciences (Shanghai, China). 5,637 and T24 (human bladder cancer cell lines) were purchased from American Type Culture Collection (ATCC). 293T, Hela and T24 were cultured in DMEM media (Invitrogen), 5,637 was maintained in RPMI-1640 media (Invitrogen). All cells were maintained by adding 10% fetal bovine serum, 1% penicillin/streptomycin (100 U/ml penicillin and 100 µg/ml streptomycin), and cultured in an atmosphere of 37◦C and 5% CO_2._

﻿**Cell Transfection**

**﻿**The plasmids were extracted by E.Z.N. A Fastfiler Endo-free Plasmid Maxiprep kits (Omega, Norcross, USA) from E. coli bacteria. Cells were transfected with a mixture of plasmids with lipofectamine 3000 (Invitrogen) according to the manufacturer’s protocols. For six-well assays, cells were transfected with the single sgRNA/dCasX construct (1 µg/μL) for 48 h before analysis.

﻿**Quantitative Real-Time PCR**

﻿According to the manufacturer’s protocol, RNAeasyTM RNA Isolation Kit (Beyotime Biotechnology, China) was used to isolate total RNA from cells transfected with different vectors. cDNA was synthesized using BeyoRTTM II cDNA Synthesis Kit (Beyotime Biotechnology, China). The mRNA expression was performed using SYBR Green qPCR MasterMix (Takara, Dalian, China), with gapdh as the control. The primers for GAPDH, GFP, c-MYC and TP53 were shown in the following sequences, with directions ranging from 5′to 3′:

GAPDH-F: TCCCATCACCATCTTCCA

GAPDH-R: CATCACGCCACAGTTTCC

GFP-F: ACGACGGCAACTACAAGACC

GFP-R: TTGTACTCCAGCTTGTGCCC

c-Myc-F: CAGCTGCTTAGACGCTGGATTT

c-Myc-R: ACCGAGTCGTAGTCGAGGTCAT

TP53-F: CCTCAGCATCTTATCCGAGTGG

TP53-R: TGGATGGTGGTACAGTCAGAGC

**Protein Isolation, Western Blot and FACS**

Protein was extracted from frozen cells by using RIPA buffer. 30μg of protein was loaded onto SDS-polyacrylamide gel electrophoresis (PAGE) gel and transferred to PVDF membranes (Bio-Rad). Then the membranes were blocked in 5% non-fat milk (blocking solution) for 1h and incubated with primary antibody overnight at 4°C. Membranes were washed with TBST three times and incubated with HRP-conjugated secondary antibodies for 1 h, and the immunoreactive bands were visualized with ECL plus Western Detection System (Bio-Rad). FACS was performed on a Fortessa flow cytometer (Becton Dickinson).

﻿**Cell Proliferation Assay**

**﻿**Cell proliferation was detected by Cell Counting Kit 8 (CCK8) assay ﻿(Transgen, China). For each well 3×10^3^ cells were seeded into 96-well plates. The cells were cultured for 24, 48, 72, and 96 h, and incubated with CCK8 at 37 ℃ for 3 h. Then the absorbance at 450 nm was measured with a microplate reader.

**Wound Healing Migration Assays**

Cells were taken from each group and inoculated in a 6-well plate at a density of 5×105 cells/well. When the cells were completely filled, a vertical line was drawn with a 10 µL spear head, and the cells were rinsed with PBS for 3 times. Photographs were taken under an inverted microscope and marked as 0 h pictures. Then, 10% fetal bovine serum DMEM medium was added for further culture for 48 h. Photographs were taken under an inverted microscope and labeled as 48 h images. The Image of 0 h was used as reference, and the software ImageJ was used to analyze the relative migration area of cells. The relative cell migration rate is equal to the migration area of the experimental group divided by the migration area of the control group as follows: wound closure = (area of gap [0 h] – area of gap [48 h])/area of gap (0 h).

**Cell Apoptosis Assay**

Cells transfected with vectors were inoculated on a 12-well-plate (2 × 10^5^ /well) with 70–80% confluency. After 48 h, the cell apoptosis was detected by the caspase-3/ELISA (enzyme-linked immunosorbent assay) assay (Hcusabio, China). The caspase-3 enzyme is a marker for inflammation and apoptosis signaling, since it can regulate the destruction of DNA or cytoskeletal proteins. Each test was performed three times.

**ELISA Assay**

Cells transfected with vectors were inoculated on a 12-well-plate (2 × 10^5^ /well) with 70–80% confluency. After 48 h, the cell was detected by the caspase-3/ELISA (enzyme-linked immunosorbent assay) assay (Hcusabio, China) and GFP ELISA kit (Abcam, USA). The caspase-3 enzyme is a marker for inflammation and apoptosis signaling, since it can regulate the destruction of DNA or cytoskeletal proteins. Each test was performed three times.

**Xenograft Model**

BALB/c nude female mice (4–5 weeks old) were randomly divided into four groups (6 per group) and housed under standard conditions. Stably transfected bladder cancer cells were subcutaneously injected into the right flanks of the mice using 5 × 10^6^ cells per mouse. Tumor volumes were measured every week. Four weeks after injection, the mice were humanely euthanized. Subcutaneous tumor tissues were isolated, and the volume and weight of the dissected tumors were measured.

The experiment procedure for AAV delivery is as below: Human bladder cancer cells (5 × 10^6^) from Matrigel (50:50) were injected subcutaneously into the lower flank of BALB/c nude female mice (4-5 weeks old) to induce tumor growth. After the tumors reached ~50 mm^3^, the mice were placed into two groups at random and the animals were treated with tail vein injections of AAV8-sgRNA-cMYC or AAV8-sgRNA-NT (1 × 10^11^vg/mice/100 μl). Tumor volumes were measured every 3-days for a period of 34 days. The mice were euthanized by decapitation and tumors were dissected.

All animal experiments were approved by the animal management committee of Peking University Shenzhen Hospital, and all experimental procedures and animal care were in accordance with the institutional ethics guidelines for animal experiments.

**Statistical Analysis**

﻿For the in vitro studies, results are shown as mean values of three independent experiments (each with three technical replicates). The experimental data were analyzed using GraphPad Prism and SPSS 20.0 (IBM, SPSS, Chicago, IL, USA). The differences between groups were analyzed by using Student’s t-test or ANOVA. The Kaplan-Meier method and log-rank test were used to assess the overall survival and disease-free survival curves. Pearson correlation analysis was used to analyze the correlations between groups. A *P* value < 0.05 was considered statistically significant.

**Supplementary Table.1**

**Maps of plasmids used in this study.**

| pHS-ACR-LW1132 | pZDonor-hU6-CasX_gRNA-backbone-GFP-sgRNA-hEF1αhEF1α-NLS-dCasX(human)-NLS-KRAB(new) terminator |
| --- | --- |
| pHS-ACR-LW1133 | pZDonor-hU6-CasX_gRNA-backbone-NC-sgRNA-hEF1α-NLS-dCasX(human)-NLS-KRAB(new)-terminator |
| pHS-ACR-LW1134 | pZDonor-hU6-CasX_gRNA-backbone-GFP-sgRNA-hEF1α-NLS-dCasX(human)-NLS-VPR-terminator |
| pHS-ACR-LW1135 | pZDonor-hU6-CasX_gRNA-backbone-NC-sgRNA-hEF1α-NLS-dCasX(human)-NLS-VPR-terminator |
| pHS-ACR-LW2408 | pZDonor-CMV-3XPAM-sgRNA-EGFP-SV40 polyA |
| pHS-ACR-LW2409 | pZDonor-3XPAM-sgRNA-TATA Box-EGFP-SV40 polyA |
| pHS-ACR-LW1136 | pZDonor-hU6-CasX_gRNA-backbone-c-MYC-sgRNA-hEF1α-NLS-dCasX(human)-NLS-KRAB(new)-terminator |
| pHS-ACR-LW1137 | pZDonor-hU6-CasX_gRNA-backbone-TP53-sgRNA-hEF1α-NLS-dCasX(human)-NLS-VPR(new)-terminator |

**Supplementary Figure 1.** The detailed profile of vectors.

pHS-ACR-LW1132 pHS-ACR-LW1133


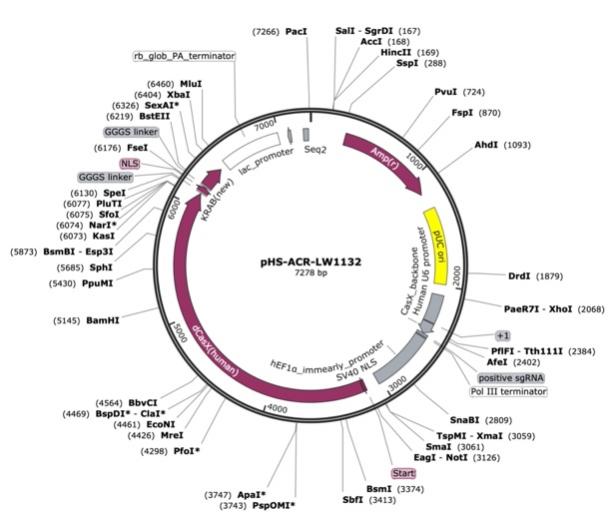

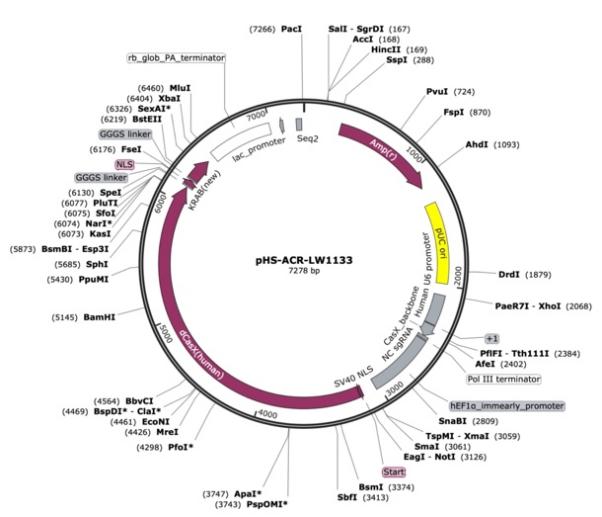


pHS-ACR-LW1134 pHS-ACR-LW1135


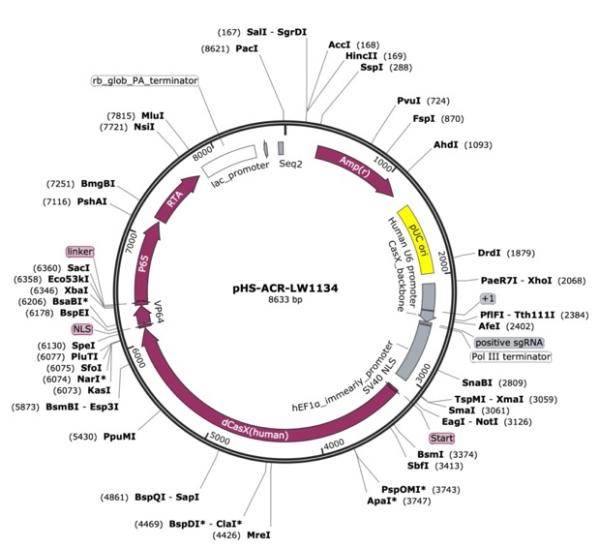

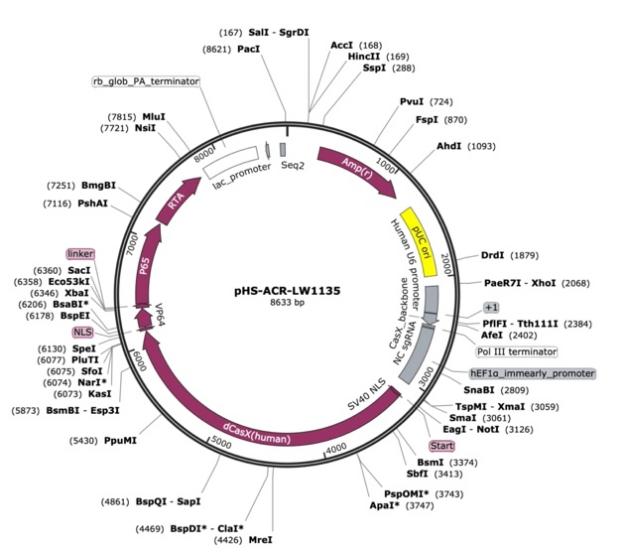


pHS-ACR-LW2408 pHS-ACR-LW2409


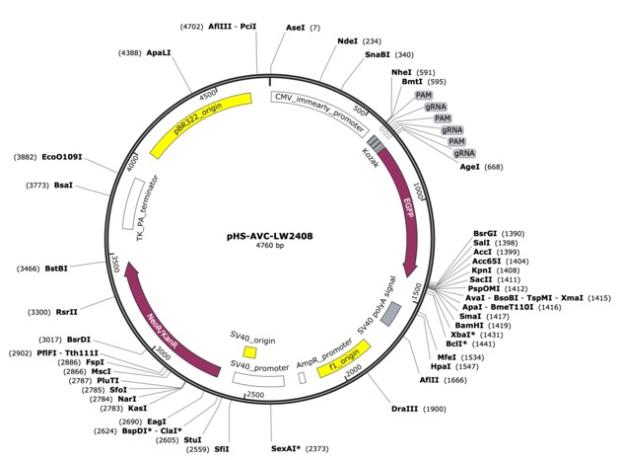

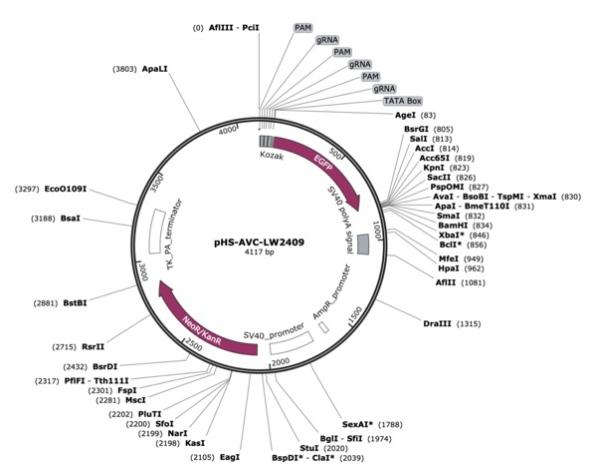


Verification of plasmid digestion


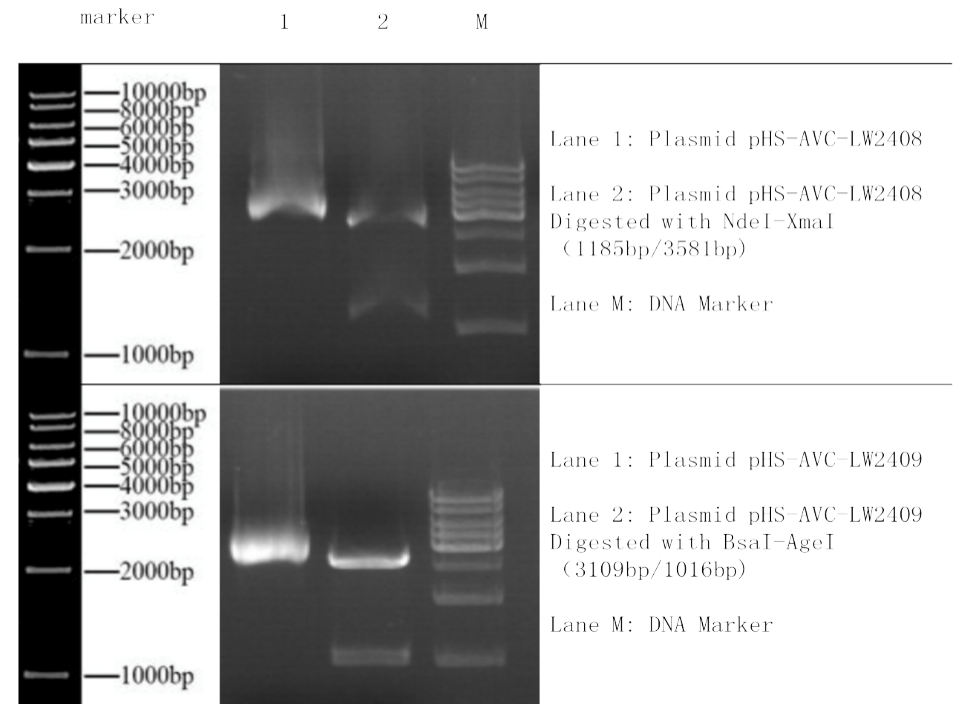


Sequencing Comparison Verification


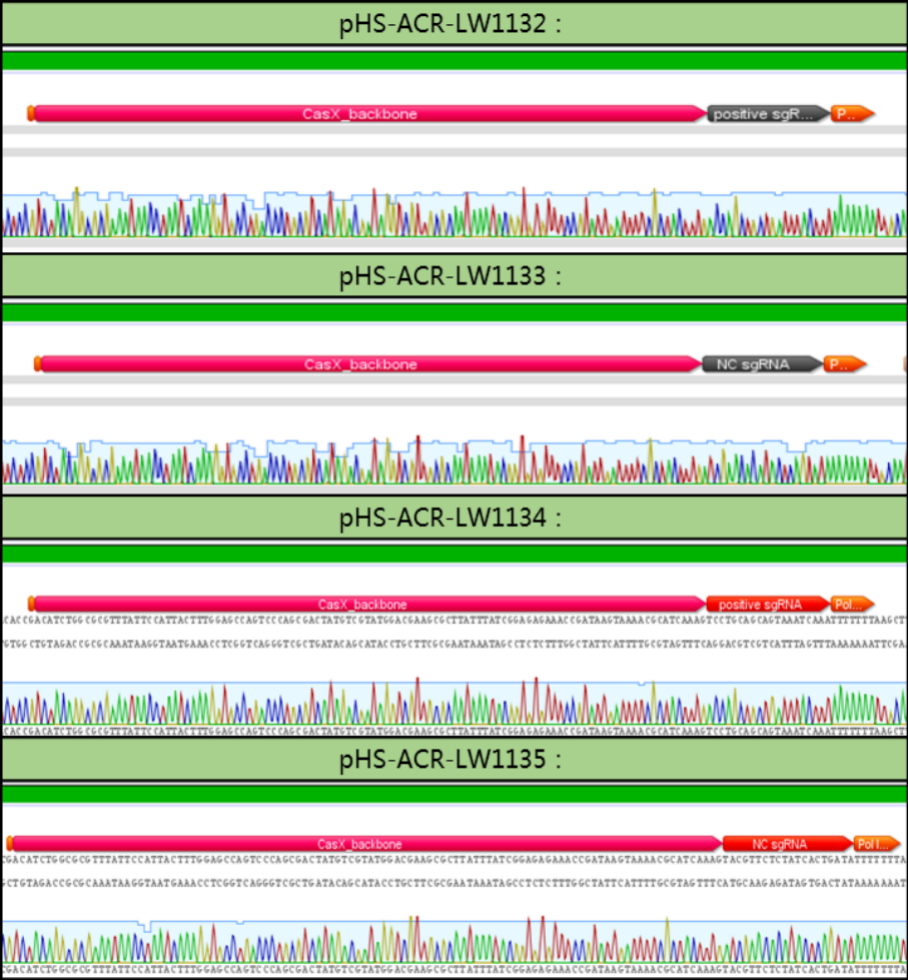


**Supplementary Figure 2.** The raw data of flow cytometry experiments for figure 1C.


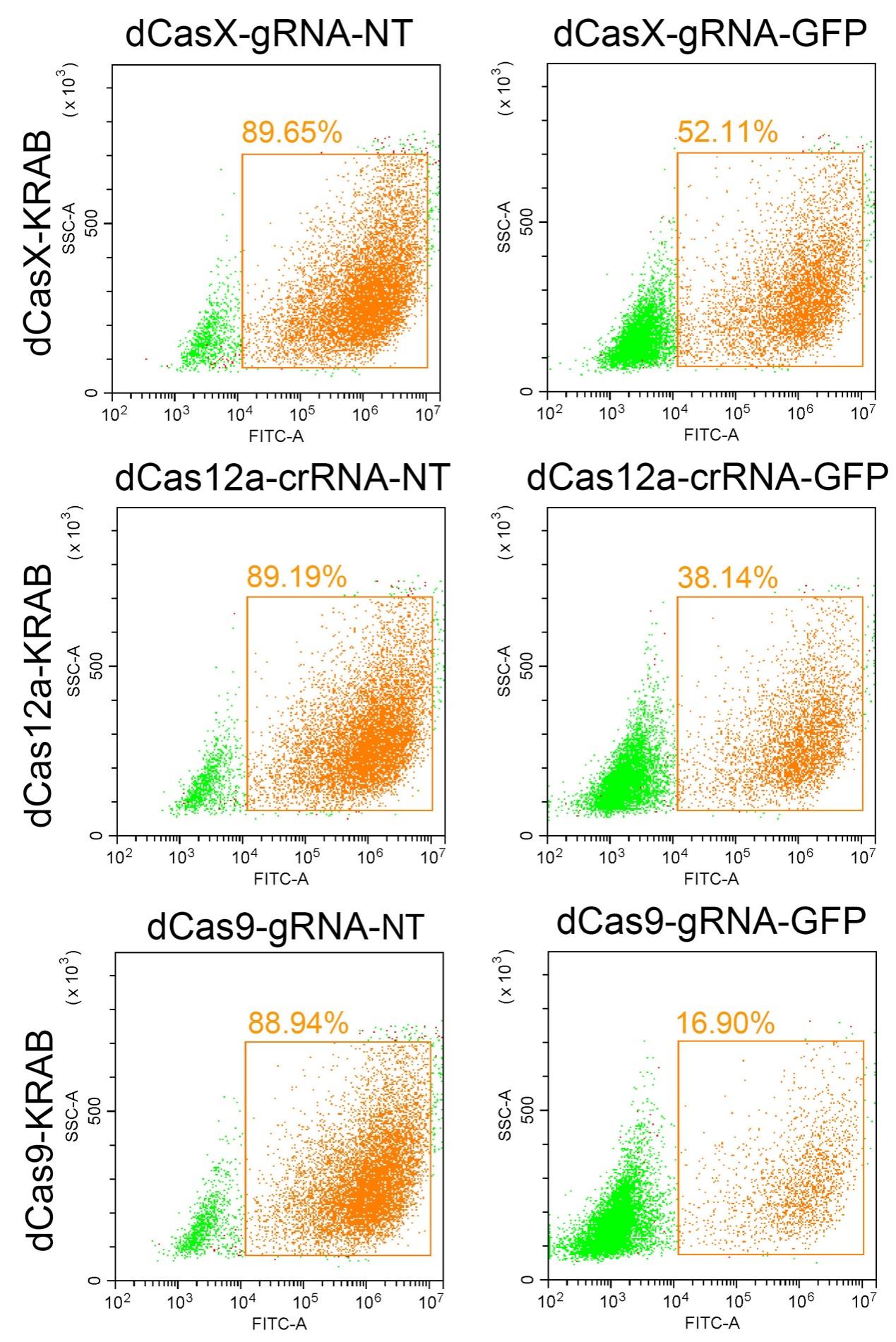


**Supplementary Figure 3.** The GFP expression in figure 1D detected by ELISA assays.


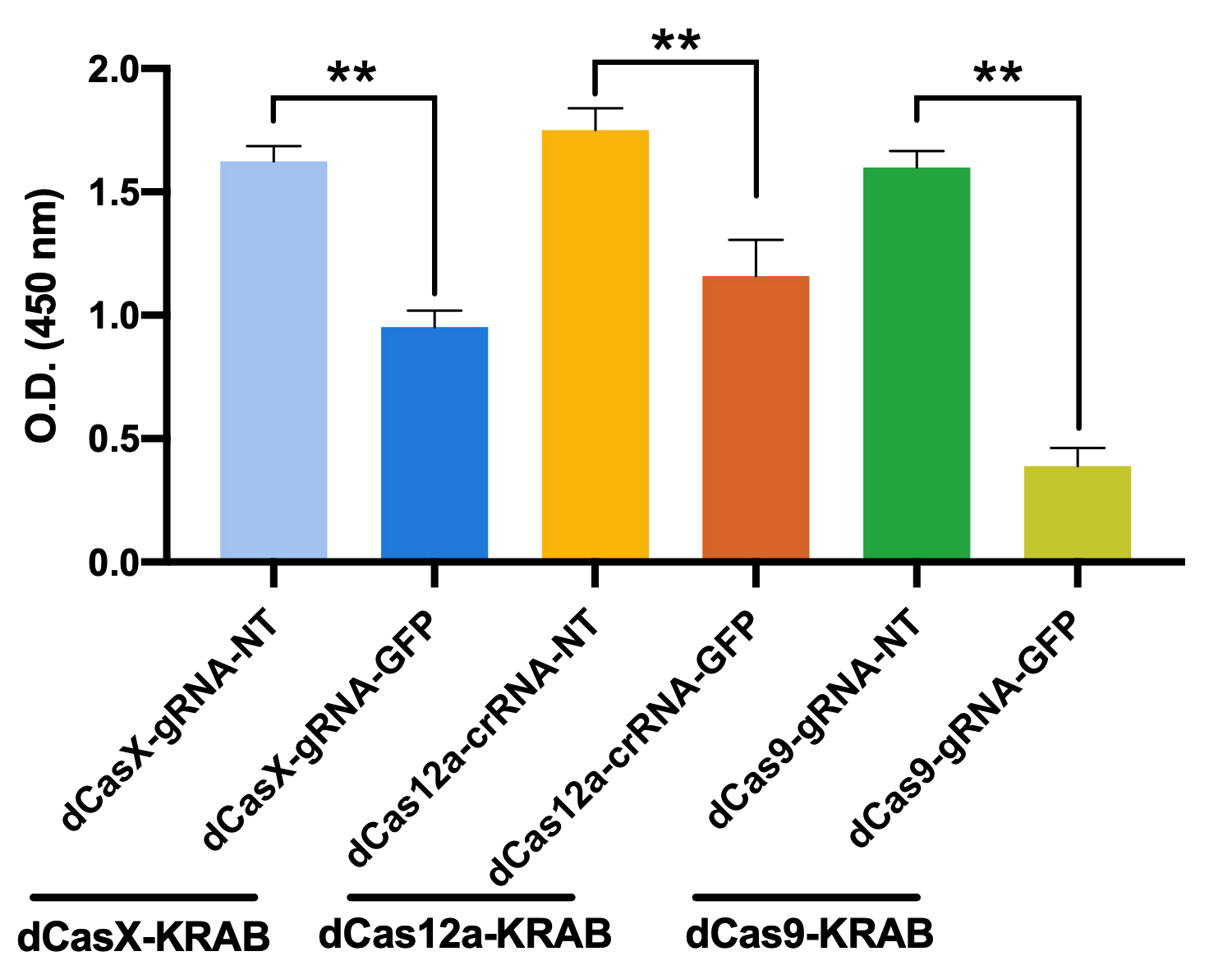


**Supplementary Figure 4.** The c-MYC expression in figure 1F detected by western blot assay.


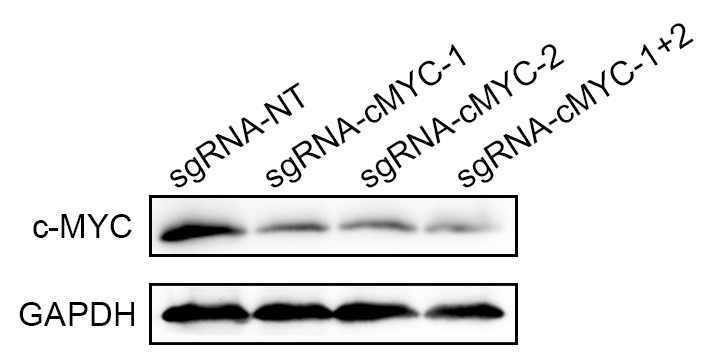


**Supplementary Figure 5.** Subcutaneous tumor model of bladder cancer cells treated with AAV-dCasX-KRAB-cMYC system and corresponding negative control.


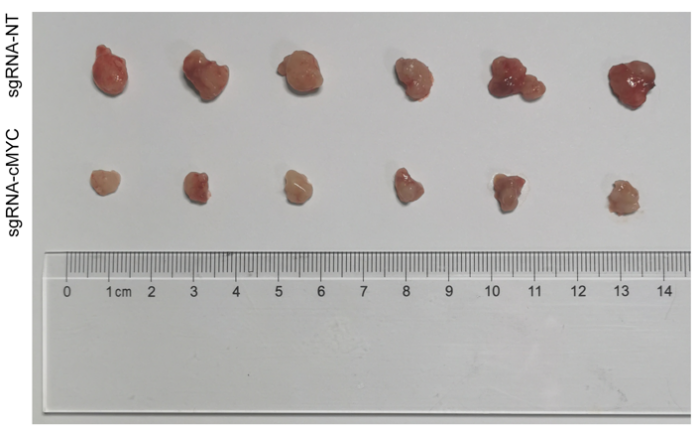

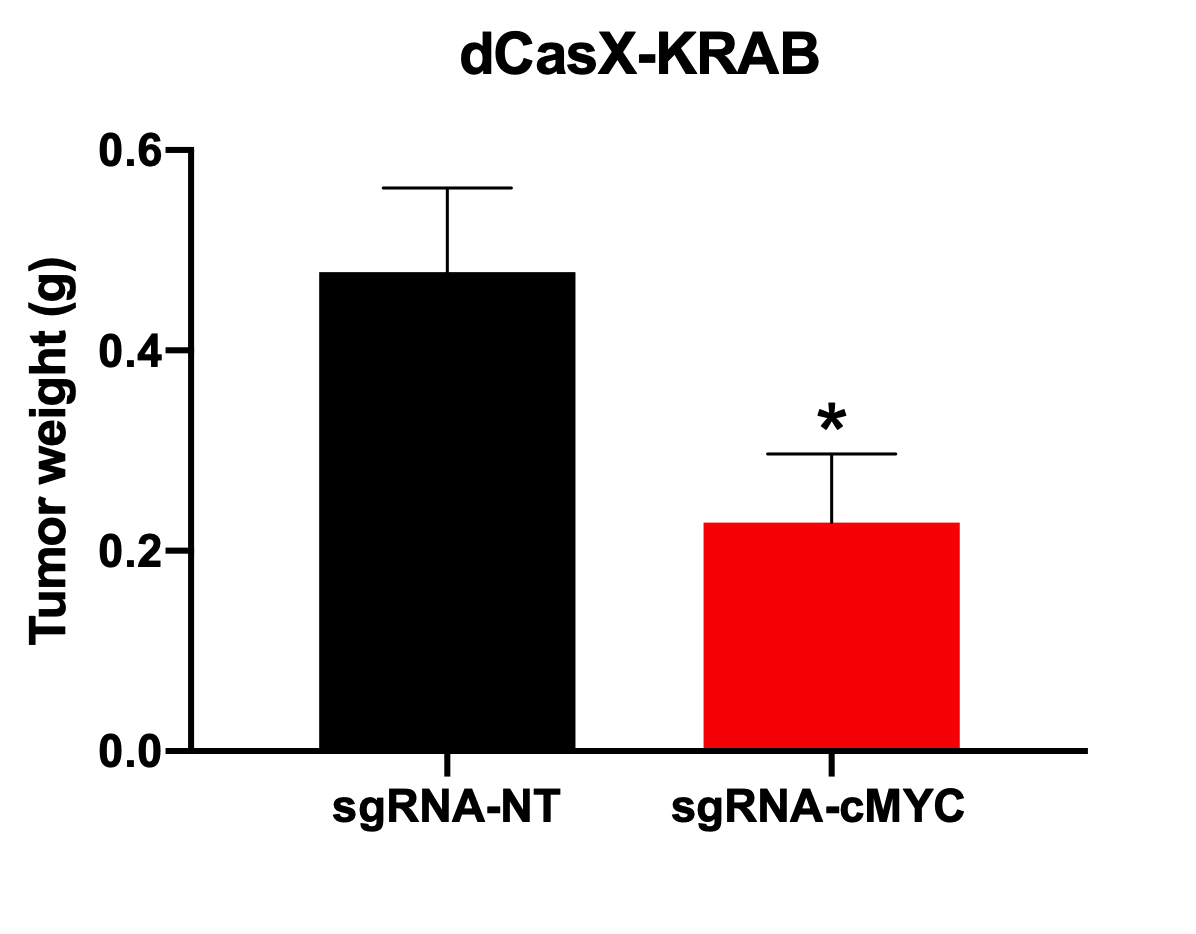


**Supplementary Figure 6.** The raw data of flow cytometry experiments for figure 3C.


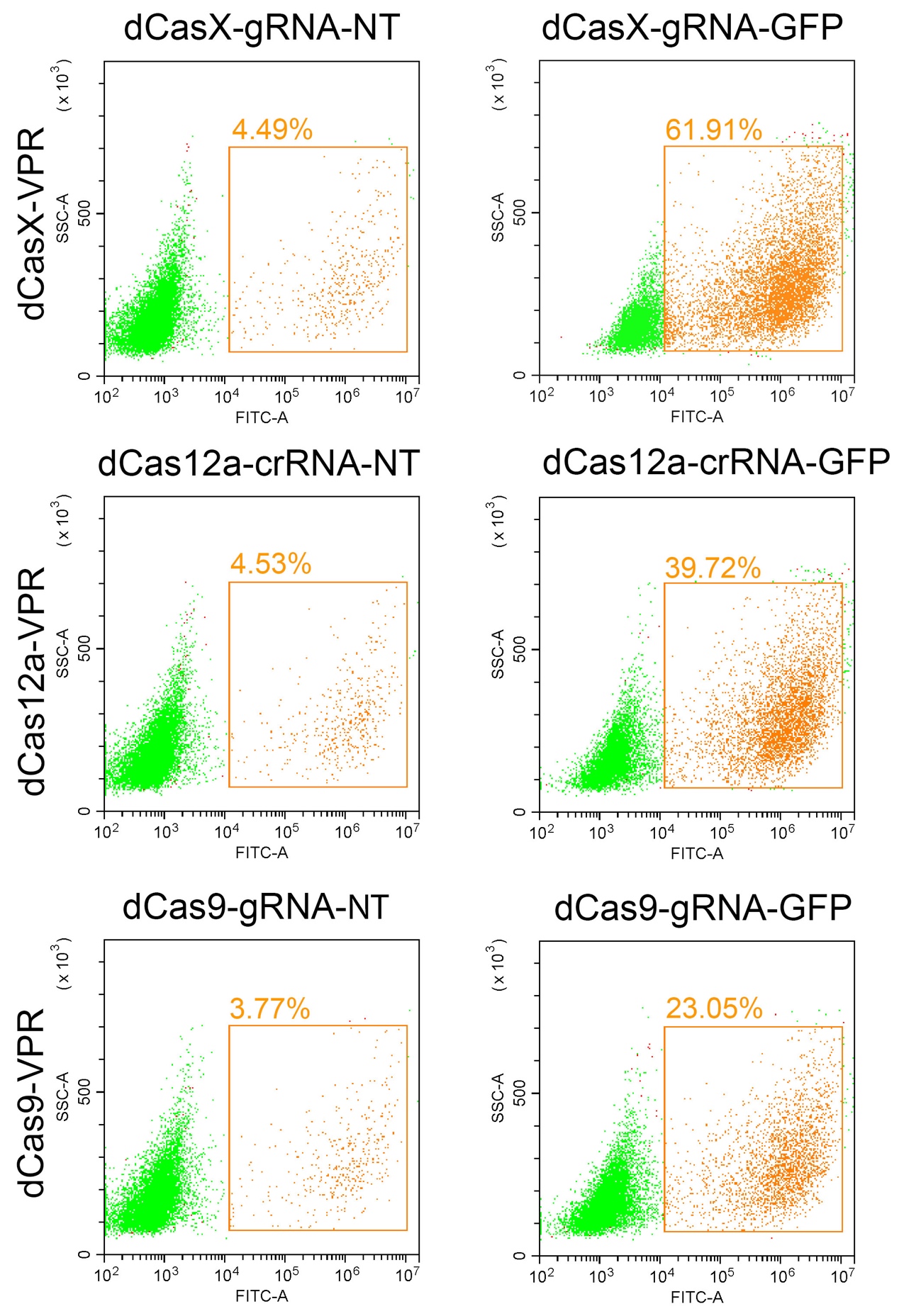


**Supplementary Figure 7.** The GFP expression in figure 3D detected by ELISA assays.


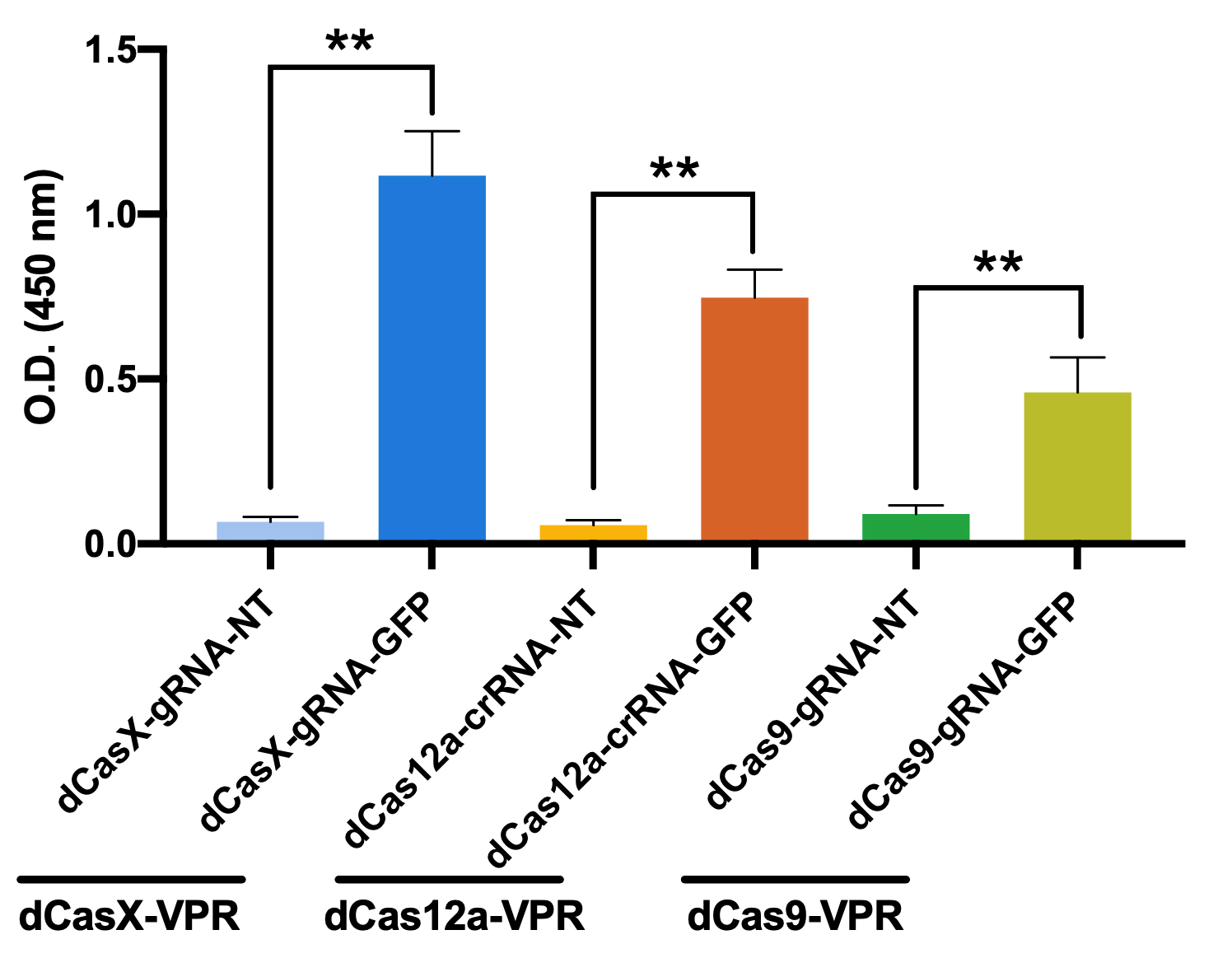


**Supplementary Figure 8.** The TP53 expression in figure 3F detected by western blot assays.


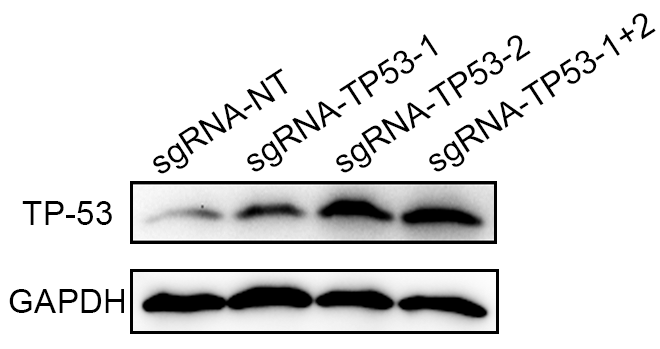


**Supplementary Figure 9.** The dCas9, dCas12a and dCasX-KRAB-based transcriptional repressors displayed RNA-guided EGFP transcriptional repression as detected by fluorescent microscopy in a stable HEK293-GFP cell line.


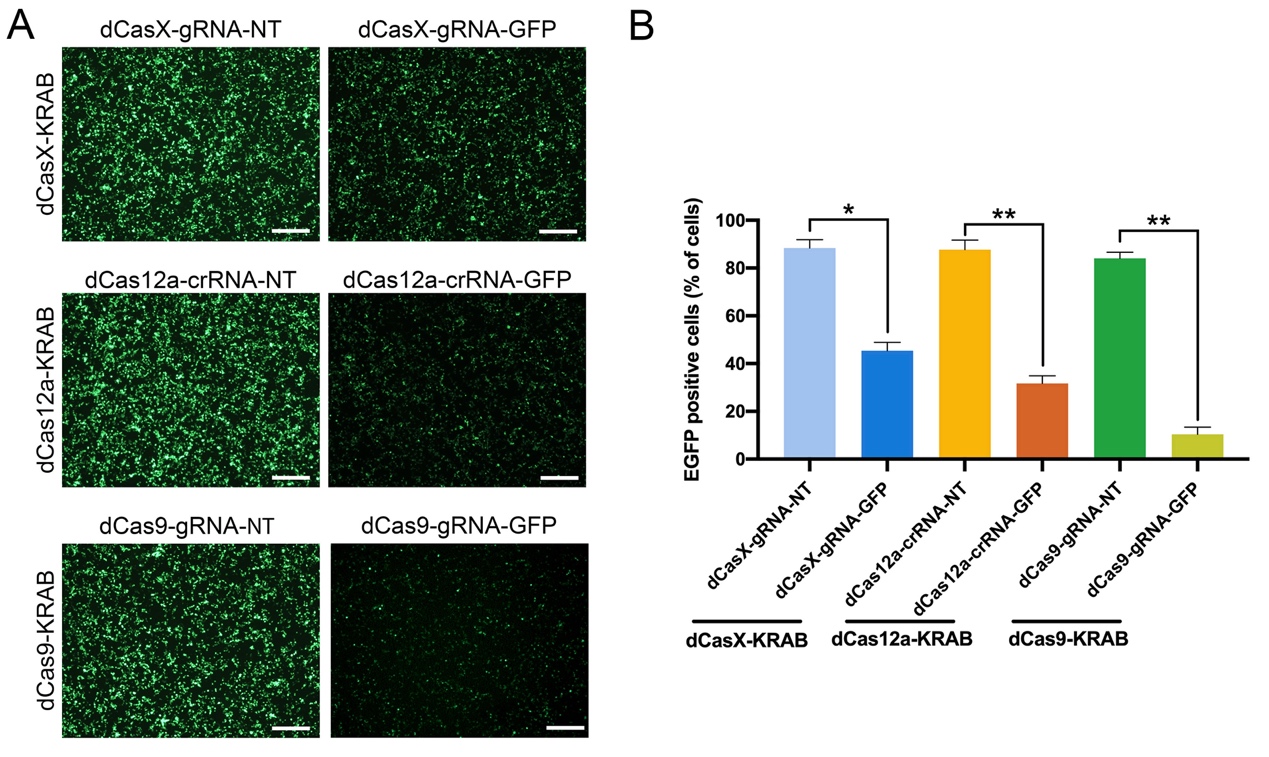


**Supplementary Figure 10.** The dCas9, dCas12a and dCasX-VPR-based transcriptional activators displayed RNA-guided EGFP transcriptional activation as detected by fluorescent microscopy in Hela cells.


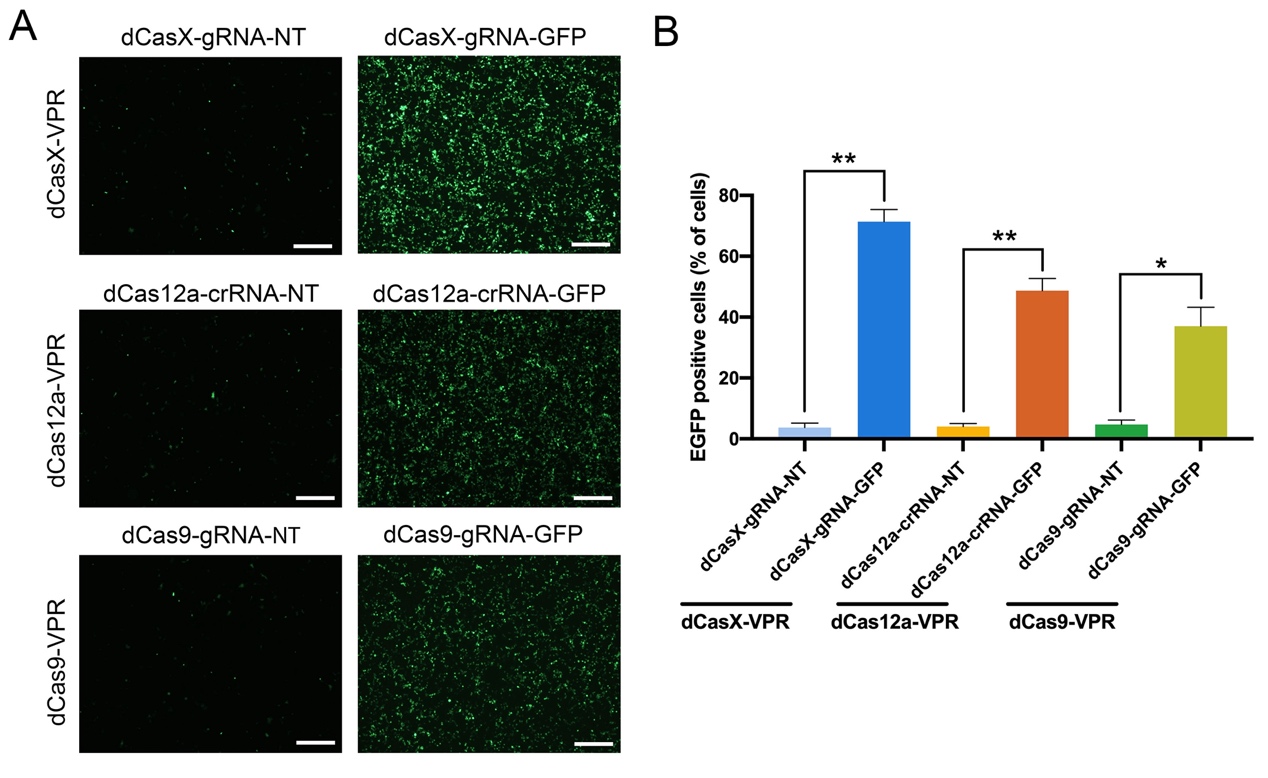


**Supplementary Table.2**

**Relative sequences used in this study.**

| Name | Synthetic Sequences |
| --- | --- |
| dCasX | GAGAAGAGGATCAACAAGATCAGAAAGAAGCTGAGCGCCGACAACGCCACAAAGCCTGTGTCCAGATCCGGCCCTATGAAAACTTTATTAGTGAGAGTGATGACAGATGATCTGAAGAAGAGGCTGGAGAAGAGGAGGAAGAAGCCCGAGGTCATGCCCCAGGTCATTAGCAACAATGCCGCCAACAACCTGAGAATGCTGCTGGATGACTACACAAAGATGAAGGAGGCCATCCTGCAGGTGTACTGGCAGGAGTTTAAGGATGATCACGTGGGCCTGATGTGCAAGTTTGCCCAGCCCGCCAGCAAGAAGATCGACCAGAATAAGCTGAAGCCTGAGATGGACGAGAAGGGCAATCTGACCACAGCCGGCTTTGCCTGTTCCCAGTGTGGCCAGCCTCTGTTCGTGTACAAGCTGGAGCAGGTGTCCGAGAAGGGCAAGGCCTACACCAACTACTTCGGCAGGTGCAACGTGGCCGAGCACGAGAAGCTGATCCTGCTGGCCCAGCTGAAGCCTGAAAAGGATAGCGATGAGGCCGTGACCTACAGCCTGGGCAAGTTCGGCCAGAGGGCCCTGGACTTTTACAGCATCCACGTGACAAAGGAGAGCACCCACCCTGTGAAGCCCCTGGCCCAGATCGCCGGCAATAGGTACGCCAGCGGCCCCGTGGGCAAGGCTCTGTCTGACGCCTGTATGGGCACCATCGCCTCCTTCCTGAGCAAGTACCAGGACATCATCATCGAGCACCAGAAGGTGGTGAAGGGCAACCAGAAGAGGCTGGAATCCCTGAGAGAGCTGGCCGGCAAGGAGAACCTGGAGTACCCCTCCGTGACCCTGCCCCCCCAGCCTCATACCAAGGAGGGCGTGGACGCCTACAATGAGGTCATTGCCAGAGTGAGAATGTGGGTGAATCTGAACCTGTGGCAGAAGCTGAAGCTGTCCAGGGACGACGCCAAGCCTCTGCTGAGACTGAAGGGCTTCCCTTCCTTTCCCGTGGTGGAGAGAAGAGAGAACGAGGTGGATTGGTGGAATACCATCAACGAGGTGAAGAAGCTGATCGACGCCAAGAGGGATATGGGCAGAGTGTTTTGGTCCGGCGTGACCGCCGAGAAGAGAAACACCATCCTGGAGGGCTACAATTACCTGCCCAACGAGAATGATCACAAGAAGAGAGAGGGCTCCCTGGAGAATCCTAAGAAGCCCGCCAAGAGACAGTTCGGCGACCTGCTGCTGTACCTGGAGAAGAAGTACGCCGGCGACTGGGGCAAGGTGTTTGACGAGGCCTGGGAGAGGATCGATAAGAAGATCGCCGGCCTGACCTCCCACATCGAGAGGGAGGAGGCCAGAAACGCCGAGGATGCCCAGTCCAAGGCCGTGCTGACAGACTGGCTGAGGGCCAAGGCCAGCTTTGTGCTGGAGAGGCTGAAGGAGATGGATGAGAAGGAGTTTTACGCCTGTGAGATCCAGCTGCAGAAGTGGTACGGCGACCTGAGGGGCAATCCCTTTGCCGTGGAGGCCGAGAACAGAGTGGTGGACATCAGCGGCTTCTCCATCGGCTCCGATGGCCACAGCATCCAGTACAGAAATCTGCTGGCCTGGAAGTACCTGGAGAATGGCAAGAGAGAGTTCTACCTGCTGATGAACTACGGCAAGAAGGGCAGAATCAGATTCACAGATGGCACCGATATCAAGAAGAGCGGCAAGTGGCAGGGCCTGCTGTACGGCGGCGGCAAGGCTAAGGTCATTGACCTGACATTCGATCCCGACGATGAGCAGCTGATCATCCTGCCCCTGGCCTTCGGCACAAGGCAGGGCAGAGAGTTTATCTGGAACGACCTGCTGAGCCTGGAGACAGGCCTGATCAAGCTGGCCAACGGCAGAGTGATCGAGAAAACTATCTATAATAAGAAGATCGGCAGAGATGAGCCTGCCCTGTTTGTGGCCCTGACATTCGAGAGAAGGGAGGTGGTGGATCCCTCCAACATCAAGCCTGTGAACCTGATCGGCGTGGCCAGGGGCGAGAATATCCCCGCCGTGATCGCCCTGACCGACCCCGAAGGCTGTCCTCTGCCCGAGTTCAAGGACTCCTCCGGCGGCCCTACCGACATCCTGAGGATCGGCGAGGGCTACAAGGAGAAGCAGAGAGCCATCCAGGCCGCCAAGGAGGTGGAGCAGAGGAGAGCCGGCGGCTACTCCAGAAAGTTTGCCTCCAAGAGCAGGAATCTGGCCGATGATATGGTGAGAAATTCCGCCAGGGACCTGTTCTACCACGCCGTGACCCACGACGCCGTGCTGGTGTTTGCCAACCTGTCCAGGGGCTTCGGCAGGCAGGGCAAGAGAACATTCATGACAGAGAGGCAGTACACAAAGATGGAGGACTGGCTGACCGCCAAGCTGGCCTACGAGGGCCTGACCAGCAAGACATACCTGAGCAAGACCCTGGCCCAGTACACCAGCAAGACCTGTTCCAATTGTGGCTTTACCATCACCACCGCCGACTACGATGGCATGCTGGTGAGGCTGAAGAAAACTTCTGATGGCTGGGCCACAACCCTGAACAACAAGGAGCTGAAGGCCGAGGGCCAGATCACATACTACAATAGGTACAAGAGACAGACCGTGGAGAAGGAGCTGAGCGCCGAGCTGGACAGGCTGAGCGAGGAGTCCGGCAATAATGATATCAGCAAGTGGACAAAGGGCAGAAGAGACGAGGCCCTGTTTCTGCTGAAGAAGAGATTTTCCCACAGGCCCGTGCAGGAGCAGTTTGTGTGCCTGGATTGCGGCCACGAGGTGCACGCCGCCGAGCAAGCTGCCCTGAATATCGCCAGAAGCTGGCTGTTCCTGAATAGCAACAGCACAGAGTTTAAGTCCTACAAGTCCGGCAAGCAGCCTTTCGTGGGCGCCTGGCAGGCCTTCTACAAGAGGAGACTGAAGGAGGTGTGGAAGCCTAACGCC |
| CasX backbone | ACATCTGGCGCGTTTATTCCATTACTTTGGAGCCAGTCCCAGCGACTATGTCGTATGGACGAAGCGCTTATTTATCGGAGAGAAACCGATAAGTAAAACGCATCAAAG |
| dCasX-gRNA-GFP | TCCTGCAGCAGTAAATCAAA |
| dCasX-gRNA-NT | TACGTTCTCTATCACTGATA |
| c-MYC sgRNA1 | GGGAGCAAACAAATCATGTG |
| c-MYC sgRNA2 | GAAACCTGGCTGAGAAATTG |
| TP53 sgRNA1 | ATCTTGGCGAGAAGCGCCTA |
| TP53 sgRNA2 | ATGTACTGAAAGCAATGAAC |
